# Factors associated with zinc utilization for the management of diarrhea in under-five children in Ethiopia

**DOI:** 10.1186/s12889-020-09541-4

**Published:** 2020-09-24

**Authors:** Achamyeleh Birhanu Teshale, Alemneh Mekuriaw Liyew, Getayeneh Antehunegn Tesema

**Affiliations:** Department of Epidemiology and Biostatistics, Institute of Public Health, College of Medicine and Health Sciences, University of Gondar, Gondar, Ethiopia

**Keywords:** Zinc utilization, Diarrhea, Under-five children, Ethiopia

## Abstract

**Background:**

Zinc has a tremendous advantage to save the life of children. It reduces the mean duration of diarrhea and mortality due to diarrhea. Besides, it reduces the severity of the initial episode, and also it prevents future diarrhea episodes. But there is low utilization of zinc for the management of diarrhea in under-five children in Ethiopia and there is a paucity of literature regarding the factors associated with the utilization.

**Method:**

The 2016 Ethiopian demographic and health survey data were used as a data source. A total weighted sample of 1228 under-five children with diarrhea within 2 weeks preceding the survey were used. A mixed-effects logistic regression analysis was done to account for the complex sample design of the data. Variables with *p*-value < 0.20 in the bivariable analysis were eligible for multivariable analysis and those variables with *p* value< 0.05 in the multivariable analysis were declared to be determinants of zinc utilization for the management of diarrhea in under-five children.

**Result:**

In this study, we found multiple determinants of zinc utilization. Mothers with formal education (adjusted odds ratio (AOR) = 1.83;95% CI; 1.30–2.58) and media exposure (AOR = 1.46;95% CI; 1.04–2.04) had higher odds of zinc utilization. But mothers from five and above household size (AOR = 0.57;95% CI; 0.39–0.82) had lower odds of zinc utilization for the management of diarrhea in under-five children.

**Conclusion:**

In our study maternal education, media exposure, and household size were determinants of zinc utilization for the management of diarrhea in under-five children. Being having a formal education and being exposed to all or either of the three media (radio, TV, and newspaper) increases the likelihood of zinc utilization while being mothers from large household size decreases the likelihood of zinc utilization. Therefore, giving special attention to those mothers with no formal education, and mothers from high family size could increase the utilization of zinc for the management of diarrhea in under-five children. Also, media campaigns regarding diarrhea management could be scaled up to potentially achieve the desired impact.

## Background

In low- and middle-income countries there is a continuing inadequate safe water and sanitation which makes diarrhea the leading cause of death among under-five children [1, 2]. Despite the availability of simple and effective treatment for diarrhea, around 8% of all deaths among under-five children were due to diarrhea in 2017. This means approximately 1300 children each day or 480,000 under-five children in a year were died due to diarrhea [3].

Since 2004, the United Nations Children’s Fund (UNICEF) and the World Health Organization (WHO) have recommended continued feeding, oral rehydration salts, and zinc supplements as management of childhood diarrhea [4]. Starting from this time zinc is used for the management of diarrhea with tremendous advantage to save the life of children. It reduces the mean duration of diarrhea by 19.7%, mortality due to diarrhea by 23%, and reduces the severity of the initial episode and subsequent diarrhea episodes in the 2–3 months following supplementation [5–7]. In addition, zinc is a trace element essential for a healthy immune system and its deficiency can make a person more susceptible to illness and disease [8, 9]. Since zinc is not stored in the body, the level of zinc is associated with dietary intake, absorption, and losses. That is zinc deficiency states may exist in children with acute diarrhea as a result of intestinal loss [10, 11]. Using zinc for the treatment of acute diarrhea helps to strengthen the immune function and intestinal structure, as well as the epithelial recovery process [12].

In addition to its effect on diarrhea, zinc supplementation had a paramount advantage to reduce respiratory illnesses (pneumonia and acute lower respiratory tract infections) and malaria among children [13–16]. For example, zinc deficiency is associated with approximately 800,000 excess deaths of under-five children throughout the world, of these 176, 000, 406, 000, and 207,000 deaths were attributable to diarrhea, pneumonia, and malaria respectively [17].

In Africa, the prevalence of good diarrhea management (using oral rehydration therapy, zinc, continued feeding with increased frequency) is low which ranges from 17% in Cote d’Ivoire to 38% in Niger [18]. Other studies in Nepal and Kenya revealed that only 15 and 18% of caregivers/mothers give zinc for the management of diarrhea for their sick child respectively [19, 20]. In Ethiopia, 46 and 33% of under-five children with diarrhea received oral rehydration therapy and zinc respectively and only 17% received a combination of these [21].

Pieces of literatures indicated that mass media exposure, maternal education, distance from the health facility, and maternal occupation are some of the factors that are associated with zinc utilization for the management of diarrhea among under-five children [20, 22–27].

Even though zinc has paramount advantages, as shown above, the utilization of zinc for the management of diarrhea in under-five children in Ethiopia is very low [21]. There is a paucity of literature regarding factors associated with this low zinc utilization for the management of diarrhea in Africa. Up to our knowledge, there was no study done regarding the factors associated with zinc utilization for the management of diarrhea in Ethiopia. Therefore, this study was aimed to assess the factors associated with zinc utilization for the management of diarrhea in under-five children in Ethiopia. The findings of this study could give insights for policymakers, health professionals, and the community in general for appropriate management of such fearful childhood illness, which is particularly common in countries with poor clinical settings like Ethiopia.

## Method

### Study design and setting

A population-based cross-sectional study was conducted in Ethiopia, which is one of the most populous African country next to Nigeria. Ethiopia is administratively classified into nine regions and two city administrations. These regions are further classified into zones, woredas, and kebeles hierarchically. Ethiopia covers 1,104,300 km^2^ with a population of around 114 million. Most (79%) of the population of Ethiopia are rural dwellers and the total fertility rate is 4.3 live births per reproductive-age women.

### Data source

The 2016 EDHS, the fourth survey conducted between January 18, 2016, to June 27, 2016, was used for this study after getting approval from the Measure DHS program through a legal request using the website https://dhsprogram.com.

### Study population and sampling procedures

**Fig. 1:**
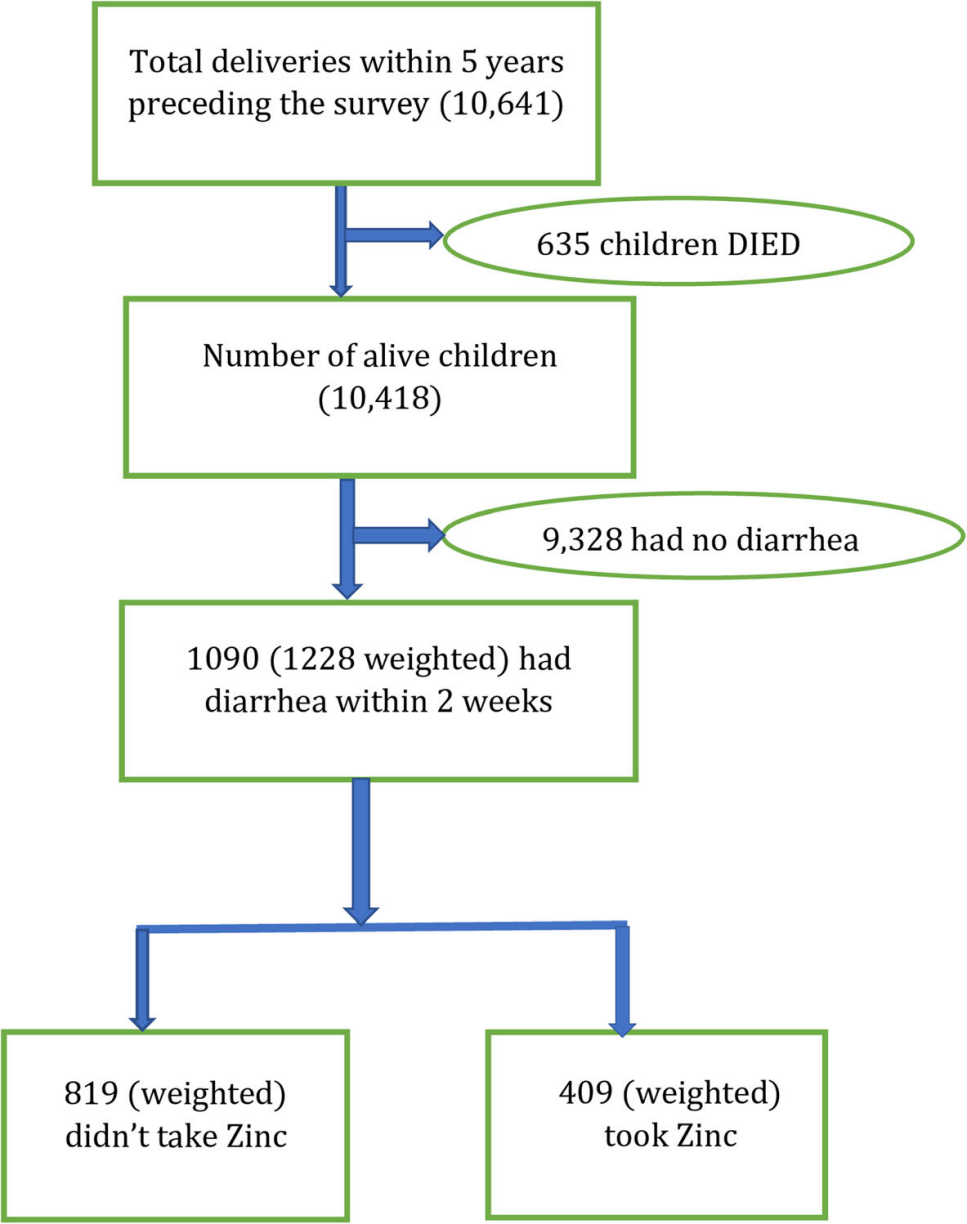
Schematic presentation of under-five children with diarrhea included in the study

The 2016 EDHS sample was selected in two stages. In the first stage, 202 clusters in urban areas and 443 clusters in rural areas were selected by using 84,915 enumeration areas from the 2007 population housing census as a sampling frame. In the second stage, a total of 28 households were selected after the household listing was carried out per each cluster. For this study, from the births data set, 10,641 total deliveries within 5 years preceding the survey were used as a target population. Several exclusion criteria were used to reach the final sample for this study. Then, a total weighted sample of 1228 living children with diarrhea within 2 weeks preceding the survey were included in the final analysis (Fig. 1).

### Study variables

The outcome variable for this study was the utilization of zinc for the management of diarrhea which was a binary outcome variable coded as “0” for No, and “1” for Yes. The independent variables included were media exposure, maternal education, maternal age, maternal occupation, marital status, religion, wealth status, health insurance coverage, parity, household size, sex of the household head, wanted the last child, perception of distance from the health facility, residence, and region. For this study, media exposure was created from three variables; frequency of watching TV, reading a newspaper, and listening radio and coded as yes if an individual was exposed to all or either of the three and no if an individual was not exposed to at least one of these. In addition, region was recoded into three categories as; small peripheral regions (Afar, Somali, Benishangul, and Gambela), large central regions (Tigray, Amhara, Oromia, and Sothern Nations Nationalities and Peoples Region), and metropolitans (Harari, Dire Dawa, and Addis Ababa), based on their geopolitical features similar to another study [28].

### Data processing and analysis

We extracted the data from EDHS 2016 and further coding and analysis were done using Stata version 14. Sampling weight was done to adjust disproportionate sampling and non-response, as well as to restore the representativeness of the sample. Descriptive statistics were done using frequency and percentage distributions. We used the mixed-effects logistic regression model approach under the generalized linear mixed model (GLMM) framework to account for the complex sample survey design. Besides, the intraclass correlation coefficient (ICC) was calculated to show whether there was a difference in zinc utilization between clusters, and model comparison was done using deviance. Both bivariable and multivariable mixed effect logistics regression model was fitted and variables with *p* < 0.2 in the bivariable analysis were eligible for multivariable analysis. In the multivariable analysis variables with *p* < 0.05 were declared to be significant factors associated with zinc utilization. Variance inflation factor (VIF) was used to test if independent variables were correlated (multicollinearity).

## Results

### Sociodemographic characteristics

The total weighted sample size used for our analysis was 1228 under-five children who had diarrhea within 2 weeks preceding the survey. The median age of the study participants was 28 (IQR = 24–33) years with the majority (29.41%) in the age group 25 to 29. A majority (62.46%) of mothers had no formal education and 94.27% were got married. Regarding media exposure, 65.27% of mothers had not exposed to either of the three media (radio, TV, and newspaper). More than two-thirds of study participants were from households with five and above household size and 88.43% of the respondents were from male-headed households. Looking at residence, 89.73% of the study participants were rural dwellers (Table 1).

**Table 1:** Sociodemographic characteristics of study participants

Variables	Weighted frequency	percentage
Maternal age		
15–19	43	3.53
20–24	263	21.44
25–29	361	29.41
30–34	311	25.29
35–39	164	13.36
40–44	68	5.51
45–49	18	1.45
Maternal education		
Have no formal education	767	62.46
Have formal education	461	37.54
Marital status		
Unmarried	71	5.73
Married	1157	94.27
Religion		
Orthodox	451	36.71
Protestant	314	25.57
Muslim	429	34.95
Others^a^	34	2.77
Maternal occupation		
Not working	649	52.82
Working	579	47.18
Wealth status		
Poor	538	43.81
Middle	268	21.79
Rich	422	34.40
Media exposure		
No	801	65.27
Yes	427	34.73
Health insurance coverage		
No	1187	96.68
Yes	41	3.32
Parity		
Primiparous	227	18.48
Multiparous	537	43.71
Grand multiparous	464	37.81
Wanted the last child		
No	370	30.11
Yes	858	69.89
Household size		
< 5	382	31.13
≥ 5	846	68.87
Sex of household head		
Male	1086	88.43
Female	142	11.57
Distance from the health facility		
Not a big problem	512	41.72
Big problem	716	58.28
Residence		
Urban	126	10.27
Rural	1102	89.73
Region		
Large central	1147	93.47
Small peripheral	55	4.45
Metropolitan	26	2.08

^a^ catholic, traditional, and other

### Random effect and model comparison

We used the intraclass correlation coefficient (ICC) to assess the random or clustering effect and deviance, others as supportive, for model comparison. The ICC in the null model was 0.185, indicating that about 18.5% of the total variation in zinc utilization was attributable to unmeasured or unmeasurable factors (random effects) and this variation was significant. Regarding model comparison, the final model (a model with lower deviance) was the best-fitted model (Table 2).

**Table 2:** Random effect and model comparison for factors associated with zinc utilization

Parameter	Null model	Final model
ICC (%)	18.5 (10.4–30.7)	12.9 (5.7–26.4)
AIC	1422.076	1381.848
LL	− 709.038	− 672.924
Deviance	1418.076	1345.848

*AIC* Akaike Information criteria, *LL* Loglikelihood

### Factors associated with zinc utilization

In the bivariable analysis variables like religion, marital status, maternal occupation, wealth status, sex of the household head, and wanted the last child had *p*-value ≥0.20 and they were not eligible for multivariable analysis. In the multivariable analysis, maternal education, media exposure, and household size were found to be significant determinants of zinc utilization for diarrhea management in under-five children at p-value < 0.05. The odds of zinc utilization during diarrheal illness was 1.83 (adjusted odds ratio (AOR) = 1.83;95% CI; 1.30–2.58) times higher among mothers who had formal education as compared to mothers with no formal education. Mothers who were exposed to either of the three media (radio, TV, or newspaper) had 1.46 (AOR = 1.46;95% CI; 1.04–2.04) times higher odds of zinc utilization for diarrhea management as compared to those mothers who were not exposed to at least one of these media. Moreover, the odds of zinc utilization among mothers from the household size of five and above were decreased by 43% (AOR = 0.57;95% CI; 0.39–0.82) as compared to mothers from a household size of below five (Table 3).

**Table 3:** Factors associated with zinc utilization for management of diarrhea among under-five children, EDHS 2016

Variables	Zinc utilization during Diarrhea		Odds Ratio (95% CI)	
	No	Yes	COR	AOR
Maternal age				
15–19	24	19	1.00	1.00
20–24	167	96	1.00 (0.48–2.08)	1.04 (0.48–2.22)
25–29	233	128	0.70 (0.34–1.44)	0.84 (0.38–1.85)
30–34	233	78	0.54 (0.26–1.14)	0.80 (0.34–1.89)
35–39	100	64	0.74 (0.34–1.59)	1.17 (0.47–2.89)
40–44	50	18	0.56 (0.22–1.43)	0.97 (0.33–2.78)
45–49	11	7	0.67 (0.16–2.74)	1.42 (0.32–6.19)
Maternal education				
Have no formal education	566	201	1.00	1.00
Have formal education	253	208	2.39 (1.79–3.21)	1.83 (1.30–2.58) **
Media exposure				
No	575	226	1.00	1.00
Yes	244	183	2.06 (1.53–2.76)	1.46 (1.04–2.04) *
Health insurance coverage				
No	797	390	1.00	1.00
Yes	22	19	1.81 (0.83–3.94)	1.58 (0.72–3.48)
Parity				
Primiparous	146	81	1.00	1.00
Multiparous	341	196	0.75 (0.51–1.11)	1.30 (0.82–2.04)
Grand multiparous	332	132	0.52 (0.35–0.78)	1.40 (0.76–2.59)
Household size				
< 5	234	148	1.00	1.00
≥ 5	586	260	0.51 (0.38–0.69)	0.57 (0.39–0.82) **
Distance from the health facility				
Not a big problem	327	185	1.70 (1.27–2.27)	1.28 (0.95–1.74)
Big problem	493	223	1.00	1.00
Residence				
Urban	62	64	1.00	1.00
Rural	757	345	0.37 (0.25–0.55)	0.71 (0.44–1.14)
Region				
Large central	770	377	1.00	1.00
Small peripheral	34	21	1.20 (0.85–1.70)	131 (0.92–1.87)
Metropolitan	15	11	2.02 (1.28–3.19)	1.43 (0.88–2.32)

*AOR* Adjusted Odds Ratio, *COR* Crude Odds Ratio, * = P < 0.05, ** = p < 0.01

## Discussion

Although zinc is included as the management option of diarrhea among under-five children, in Ethiopia a small proportion of women with under-five children used zinc for the management of diarrhea. In this study, we assessed the factors associated with zinc utilization for the management of diarrhea in under-five children using a mixed-effects logistic regression model. We found maternal education, media exposure, and household size as determinants of zinc utilization during diarrheal illness in under-five children.

The odds of zinc utilization during diarrheal illness in under-five children were higher among children whose mothers had formal education as compared to their counterparts. This is in line with a study done in North-Western Nigeria on the use of zinc in under-five children with diarrhea [25]. This might be due to educated mothers might have awareness regarding their child’s health and they might be active to take their sick child to the nearest health facility. This, in turn, might create an opportunity for mothers to get advice from health professionals and give the recommended or the prescribed medication including zinc for their sick child.

Consistent with studies done in Bangladesh [24], Nepal [20], India [27], and Ghana [23], in this study the likelihood of using zinc for the management of diarrhea in under-five children was higher among mothers who had media exposure as compared to mothers with no media exposure. This might be because of advertising through both television and radio regarding zinc syrup or tablets are the quickest media channels in terms of creating or disseminating information on the benefits of zinc in the country. Also, even though in Ethiopia reading newspapers is not common, reading newspapers and posted materials around the pharmacy might have a great advantage for understanding zinc as an important component for the management of diarrhea in under-five children. These all can result in a great opportunity for creating awareness for caregivers/mothers to give this very important micronutrient for her sick child.

Moreover, mothers with higher household members (five or above) had lower odds of giving zinc to their child with diarrhea as compared to mothers from less than five household members. This might be because mothers with larger household sizes might be busy by many routine activities and they are unable to give appropriate care for her sick child. In addition, mothers from large household sizes might have parenting stress which might affect appropriate child feeding practices and health-seeking behavior to get treatment if their child becomes sick.

The clinical and public health implication of this study is to enhance zinc utilization for the management of diarrhea in under-five children by identifying the factors attributed to the low utilization. Therefore, considering and taking special attention of factors that favor the low utilization of zinc for the management of diarrhea among under-five children such has mothers having no formal education, mothers have no exposure to media, and mothers from large household size could increase zinc utilization.

This study had both strengths and limitations. The strength of this study is just it is based on nationally representative data with appropriate analysis techniques. This study is also the first to assess the determinants of zinc utilization in under-five children during diarrheal illness in Ethiopia. Since there was a paucity of literatures/studies done before, we have got difficulty in discussing our findings by comparing it with other studies. Also, mothers/caregivers were asked to remember “whether she gave zinc while her child got diarrhea” there may be a lapse of memory or recall bias. Moreover, due to the cross-sectional nature of the data clear temporality (cause and effect relationship) was not established between the dependent and independent variables.

## Conclusion

We found multiple determinants of zinc utilization for the management of diarrhea in under-five children. These factors are; maternal education, media exposure, and household size. Being having a formal education and media exposure increases the odds of zinc utilization and being mothers from larger household sizes decreases the odds of zinc utilization for the management of diarrhea in under-five children. Therefore, media campaigns regarding diarrhea management options could be scaled up to potentially increase zinc utilization. Also, giving special attention to those mothers with no formal education and mothers from large household sizes could increase the utilization of zinc for the management of diarrhea in under-five children.

## Data Availability

We included all result-based data within the manuscript and the data set can be accessed online from www.measuredhs.com/data.

## Abbreviations

AOR: Adjusted Odds Ratio
AIC: Akaike Information criteria
CI: Confidence Interval
EDHS: Ethiopian Demographic and Health Survey
DHS: Demographic and Health Survey
GLMM: Generalized Linear Mixed Model
ICC: Intra Class Correlation
LL: Loglikelihood
UNICEF: United Nations Children’s Fund
VIF: Variance Inflation Factor
WHO: World Health Organization
